# Diagnostic Application of Postmortem Cardiac Troponin I Pericardial Fluid/Serum Ratio in Sudden Cardiac Death

**DOI:** 10.3390/diagnostics11040614

**Published:** 2021-03-30

**Authors:** Diana Hernández-Romero, María del Rocío Valverde-Vázquez, Juan Pedro Hernández del Rincón, José A. Noguera-Velasco, María D. Pérez-Cárceles, Eduardo Osuna

**Affiliations:** 1 Department of Legal and Forensic Medicine, Faculty of Medicine, Regional Campus of International Excellence “Campus Mare Nostrum”, Biomedical Research Institute (IMIB), University of Murcia, 30100 Murcia, Spain; rociovv1014@gmail.com (M.d.R.V.-V.); mdperez@um.es (M.D.P.-C.); eosuna@um.es (E.O.); 2 Institute of Legal Medicine, 30003 Murcia, Spain; jphrincon@um.es; 3 Clinical Analysis Service, Hospital University “Virgen de la Arrixaca”, El Palmar, 30120 Murcia, Spain; josea.noguera@gmail.com

**Keywords:** cardiac troponin I, pericardial fluid, serum, myocardial infarction, postmortem diagnosis

## Abstract

In approximately 5% of unexpected deaths, establishing a conclusive diagnosis exclusively on the basis of anatomo-pathological findings in a classic autopsy is difficult. Postmortem biomarkers have been actively investigated as complementary indicators to help to reach valid conclusions about the circumstances of death. Several studies propose either the pericardial fluid or peripheral veins as a location for troponin determination, but the optimum sampling site is still a matter of debate. Our objective was to evaluate the association between the ratio of troponin values in the pericardial fluid and serum (determined postmortem) and the diagnosis of acute myocardial infarction (AMI) in the context of sudden cardiac death. We included 175 forensic cases. Two groups were established: AMI deaths (48; 27.4%) and the control group (127; 72.6%). The cardiac Troponin I (cTnI) values in the pericardial fluid and the troponin ratio were found to be associated with the cause of death. Univariate regression analyses showed that both age and the cTnI ratio were significantly associated with the diagnosis of AMI death. In a multivariate analysis, adjusting for confounding factors, the age and cTnI ratio were independent predictors of death from myocardial infarction. We performed a receiver operating characteristic (ROC) curve for the cTnI ratio for AMI death and selected a cut-off point. Our biomarker was found to be a valuable and highly effective tool for use in the forensic field as a complementary method to facilitate diagnosis in nonconclusive autopsies.

## 1. Introduction

The postmortem pathological study of unexpected death is diagnostic in a significant percentage of cases; however, up to 5% of rapid and unexpected deaths yield negative autopsies that do not facilitate a conclusive diagnosis [1]. Complete necrosis of myocardial cells requires 2–4 h of permanent ischemia [2]. Hence, establishing a conclusive diagnosis in sudden death exclusively on the basis of the anatomo-pathological findings of a classic autopsy can be difficult [3], especially with the short survival period from the onset of symptoms.

Postmortem biomarkers have been very actively investigated as complementary indicators to help reach valid conclusions about the circumstances of death [4]. However, biochemical analyses in the corpse can be affected by postmortem changes, in which many compounds undergo important transformation processes, making the postmortem interval a parameter that influences the usefulness of biochemical markers [5,6]. This being said, it was reported that in cases with an early sample extraction, many of the biochemical determinations show utility in the forensic field [7]. For the postmortem diagnosis of myocardial infarction, apart from the serum, the most suitable body fluids are peripheral blood, pericardial fluid and vitreous humor [8,9,10].

Biomarkers are able to passively diffuse into the pericardial fluid, being detectable in higher levels in this fluid than in serum [11,12].

Various studies have been carried out in forensic practice to understand the usefulness of determining biochemical markers of myocardial damage [7,13,14]. The determination of troponins acquires a greater diagnostic validity when associated with other biochemical parameters whose relevance is especially accepted in the clinical practice, such as B-type natriuretic peptide (BNP) or the N-terminal portion of the pro-B-type natriuretic peptide (NT-proBNP) [15]. Furthermore, some biomarkers that are used for the diagnosis of acute myocardial infarction (AMI) may also be present in serum due to musculoskeletal injury. Moreover, ischemia without necrosis has also been proposed as a mechanism for troponin release [16].

In the case of troponins, several studies propose either the pericardial fluid [17] or different peripheral veins [18,19] as the best sampling location for troponin determination in the forensic context; hence, the preferred sampling site is still under intense debate. The advantages of determining the ratio between pericardial fluid and peripheral blood values were evaluated for several cardiac biomarkers in the postmortem diagnosis of myocardial injury [12,20]. However, to our knowledge, no studies have been performed on troponin to evaluate the diagnostic performance of the ratio in the context of forensics in sudden death cases.

Hence, we hypothesized that this ratio of cardiac troponin I (cTnI) values in the serum and pericardial fluid may shed light on the sampling site debate, thus retaining the advantages of the both locations. The main objective of the present study was to evaluate the postmortem determination of the ratio of cTnI values in the pericardial fluid and serum for the diagnosis of AMI in the context of sudden death. We propose the cTnI ratio as a complementary tool in postmortem diagnoses that can be used together with anatomo-pathological findings.

## 2. Materials and Methods

### 2.1. Study Design

We carried out an observational, retrospective, double-blind study. Forensic cases from routine autopsies were consecutively included at the Institute of Legal Medicine of Murcia throughout 2019. Inclusion criteria were: (i) age ≥ 18 years old; (ii) confirmed cause of death after autopsy, both by macroscopic and microscopic examinations, such as AMI, asphyxia, chest trauma or other natural causes of death; (iii) postmortem interval less than 24 h, to avoid possible interference due to autolysis processes and (iv) blood samples collected without hemolysis. The exclusion criterion was not meeting at least one inclusion criterion. Of the 523 autopsies, 175 final forensic cases were included and classified as AMI, asphyxia, chest trauma or natural death. To minimize the possible dispersion of data due to the autolysis phenomena, the corpses were preserved in a cold room until the autopsy was performed. In all cases, the existence of a survival time was known, according to information from the anamnesis of the people related to the deceased, and from the information obtained in the ocular inspection and the removal of the body. For the study of the data, two diagnostics groups were established: one group of deaths by AMI according with the Fourth Universal Definition of Myocardial Infarction [20] and another group of noncardiac etiology or a control group. The study was approved by the Ethics Committee of the Institute of Forensic Medicine and the Ethics Committee of the University of Murcia (approval number 2323/2019).

### 2.2. Procedure for Extraction of Biological Fluids

During the autopsy, up to 6 mL of peripheral blood and 10 mL of pericardial fluid were collected for each patient from the femoral vein and pericardial sac, respectively, with a sterile syringe. The samples were allowed to clot and centrifuged within 2 h after extraction. Samples were stored for preservation at a temperature of −80 °C until the biochemical batch analysis was carried out.

### 2.3. Biochemical Determinations

Ultra-sensitive troponin I (hscTnI) concentrations were determined using the “STAT High-Sensitive Troponin-I assay” (Architect-Abbott) assay (Abbott Diagnostic, Chicago, IL, USA), which uses mouse monoclonal antibodies that recognize two epitopes in human hs-cTnI. It demonstrated a detection limit of 1.5 ng/L, a 99th percentile of <26 ng/L, and a coefficient of variation of 10% at 3 ng/L.

### 2.4. Statistical Analysis

Each categorical variable is expressed as the frequency (percentage) of cases. Continuous variables were tested for normal distribution using the Kolmogorov–Smirnov test. The normal distributed continuous variables are shown as mean ± SD, and those that were nonparametrically distributed are shown as median (interquartile range). Differences in cTnI values between groups were assessed using the Mann–Whitney U “t” or Kruskal–Wallis tests (as appropriate). Correlation was performed between two continuous variables using the Spearman test. A two-side probability value of *p* < 0.05 was considered statistically significant. Multivariate analysis by linear regression was used to identify the factors associated to cTn I variables. Variables with *p* < 0.15 in the univariate analysis were included into the multivariate regression model. Statistical analyses were carried out with SPSS version 24.0 software for Windows (IBM SPSS Statistics, Inc., Chicago, IL, USA).

## 3. Results

We included a total of 175 forensic cases (82.3% men; 51.3 ± 18.9 years) in the Murcia Institute of Legal Medicine. In 47 (26.9%) cases, cardiopulmonary resuscitation was performed. We included cases with a postmortem interval of less than 24 h, the average being 8.3± 4.9 h. Two groups were established to divide AMI deaths (48; 27.4%) from the control group without previous cardiovascular pathology (127; 72.6%), consisting of other causes of death including traumatic (39; 22.3%), asphyctic (55; 31.4%) and natural deaths (33; 18.9%) (Table 1). We observed differences in age among groups (*p* > 0.001; Table 1). No significant difference was observed in race or ethnicity, or in weight or height among groups. cTnI was determined in both the serum and pericardial fluid and the ratio between pericardial and serum troponin was also calculated. We performed post-hoc analyses and obtained an 89% power calculation for our study. Correlation analyses demonstrated that serum (r-value: 0.21; *p*-value: 0.006) and the ratio (r-value: −0.15; *p*-value: 0.043) values were correlated with PMI, whereas this correlation was not found for the pericardial cTnI values (r-value: 0.03; *p*-value: 0.655). When additional linear regression analyses were performed, none of the three variables were demonstrated to be influenced by the postmortem interval of death (data not shown).

### 3.1. Association of Cardiac Troponin I with the Diagnosis of Death

We evaluated the association of cTnI with the causes of death. Significant differences in cTnI values were observed when comparing the different causes of death (Table 2). Furthermore, when the association between cTnI variables and the cause of death was analyzed, both cTnI values in the pericardial fluid and the troponin ratio were associated with the cause of death (*p* <0.05; Table 2).

### 3.2. Study of Cardiac Troponin I as a Predictor of Diagnosis of Death from AMI

We studied cTnI levels in serum, pericardial fluid and the calculated ratio to establish their predictive utility in the postmortem diagnosis of AMI. cTnI levels in the pericardial fluid, and the ratio levels, showed significant correlations with the diagnosis of AMI death. As for serum cTnI, only a borderline significance was demonstrated (Table 3). Moreover, both cTnI in the pericardial fluid and the ratio showed significantly different values between the group of AMI deaths and the control group (Table 3). Therefore, we decided to select the cTnI ratio, instead of serum levels, due to the superior performance regarding its correlation with AMI diagnosis and differentiation into diagnostics groups.

Thereafter, we aimed to study the cTnI ratio as a predictive biomarker for diagnosing AMI death. In this sense, univariate regression analyses showed that both age and the cTnI I ratio were significantly associated with the diagnosis of AMI death. In a multivariate analysis, adjusting for confounding factors, age and cTnI ratio remained as independent predictors of death from myocardial infarction (Table 4).

### 3.3. Search for a Cut-Off Point for the Ctni Ratio in the Diagnosis of AMI Death

**Figure 1:**
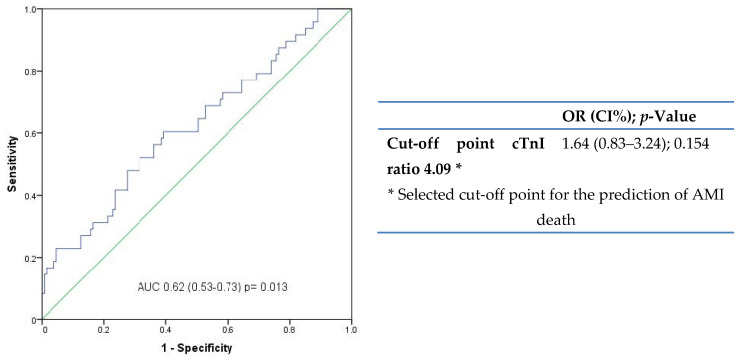
Receiver operating characteristic (ROC) for the evaluation of the cTnI ratio related to AMI death, and the selection of the cTnI ratio cut-off point in the prediction of AMI death (lineal regression).

We investigated a possible cut-off point for the cTnI ratio values to aid in the postmortem diagnosis of AMI by providing a reference diagnostic value. We plotted receiver operating characteristic (ROC) curves related to AMI death (AUC: 0.621 ± 0.048 (CI95%: 0.527–0.716); *p*= 0.013; Figure 1) and established a cut-off point for the cTnI ratio of >4.09. The regression analysis, however, did not demonstrate a significant association with AMI death (OR (95% CI): 1.64 (0.83–3.24), *p* = 0.154).

## 4. Discussion

In the postmortem diagnosis of AMI, numerous biomarkers have been studied, such as BNP, NTproBNP and cardiac troponins [5,11,15,21]. These biomarkers appear earlier and in a higher concentrations in the pericardial fluid than in peripheral blood, due to their contiguity with the myocardium and its passive diffusion during the early postmortem period [22]. In advancing the search for more precise cardiac biomarkers, we hypothesized that the determination of the ratio between cTnI values expressed in pericardial fluid and in peripheral blood may be useful in the postmortem diagnosis of AMI deaths. In our study, we demonstrated, for the first time, that the cTnI ratio between pericardial fluid and serum presented statistically better characteristics than cTnI in the serum or pericardial fluid alone. In addition, it was an independent biomarker in the diagnosis of AMI deaths, as demonstrated in our multivariate analysis, after adjusting for confounder factors.

### 4.1. cTnI Ratio is Associated with the Cause of Death

In previous studies [12,23], we demonstrated the postmortem diagnostic utility of cTnI in the pericardial fluid and serum in AMI deaths. Moreover, Cina et al. [24] found a correlation between cTnI concentrations and the cause of death. Herein, we show that cTnI values in the pericardial fluid and the cTnI ratio levels were significantly different among groups separated by cause of death. Highest cTnI ratio values were shown for AMI deaths, followed by asphyctic, traumatic and deaths by other natural causes. These differences were not significant for serum cTnI values. Similarly, cTnI values in the pericardial fluid and cTnI ratio values were significantly associated with the cause of death, which, again, was not the case for serum values. It should be kept in mind that biomarkers in serum could undergo autolysis phenomena that may result in reduced specificity for cardiac pathology in this medium. This is due to their release into the bloodstream, which is also sometimes the case in musculoskeletal injuries.

### 4.2. cTnI Ratio Is an Independent Predictor of AMI Death

Ooi et al. [25] reported a correlation of CK-MB, cTnI and cTnT values with the presence of pathological findings suggestive of heart damage, in autopsies of patients without symptoms of AMI. Both cTnT and cTnI showed diagnostic utility in deaths from AMI and highlight their high diagnostic specificity. cTnI presented a very high negative predictive value, making it the quintessential marker for ruling out cardiac damage if its concentration is not altered [15]. Herein, we also found that both cTnI in the pericardial fluid and the ratio values correlated significantly with AMI death. In addition, both cTnI variables showed significantly different values between the control group and the group of AMI deaths. These results are in agreement with previous studies that show greater changes in the serum than in the pericardial fluid. These are probably caused by postmortem changes that are more sensitive at the serum level, such as redistribution or hemoconcentration [5,15,24]. In addition, these biomarkers diffuse in the pericardial fluid more rapidly due to its contiguity with the myocardium. They also do so in a more stable way since the autolysis processes take place in a later postmortem interval, due to the protective characteristics of the pericardial sac [22]. Moreover, the interest in determining the ratio between the pericardial fluid and serum values of certain biomarkers was previously reported [14,23]. On the basis of the cTnI ratio results, and the advantage of it being a normalized biomarker that minimizes the heterogeneity of pericardial and serum determinations, we selected the cTnI ratio as the preferred biomarker. In addition, we constructed ROC curves and found significant AUC values. However, our selected cut-off point of 4.09 did not reach statistical significance for AMI prediction. Our results represent an important contribution to the forensic field, in which the diagnostic validity of the cTnI ratio remains untested. According to our results, the cTnI ratio shows relevant advantages over serum cTnI in this context.

### 4.3. Limitations

In our study, various circumstances limiting the routine use of these biomarkers were described. We must remember that, in our study, only forensic cases with a postmortem interval of less than 24 h were included in order to limit the occurrence of autolysis phenomena. Hence, the application of our results in cases with higher postmortem intervals needs to be proved. Furthermore, the corpses were kept in cold rooms until the autopsy was carried out. To date, physical methods are routinely used to determine the date of death, always framed in a postmortem interval. Hence, these limiting factors result in the need for an early postmortem interval, the rapid collection of samples, and the preservation of the samples at the appropriate temperature, all in order to avoid autolysis phenomena that may interfere with the validity of the results. The study only included patients for whom a cause of death was eventually diagnosed; hence, its applicability in a case in which the cause of death is not able to be ascertained based on history or autopsy is uncertain. Finally, it is necessary to establish universal cut-off points as referential values to determine the etiological diagnosis.

### 4.4. Future Directions

The use of our proposed biomarkers is ideal in the context of biochemical determinations as complementary techniques to help the classical autopsy achieve a more accurate diagnosis. However, we also think that the underlying pathophysiology of biomarker kinetics and distribution among corporal fluids may be of interest in clinical medicine and the diagnosis of cardiac biomarkers in sudden death. Larger cohorts are needed to validate our results in less controlled scenarios. This will be a step towards the establishment of universal cut-off points as referential values in forensic diagnosis.

## 5. Conclusions

In summary, we demonstrated that postmortem biomarkers were useful in the diagnosis of the cause of death. Frequently, the pathological analysis did not reveal conclusive information and complementary tests become necessary. In conclusion, we proposed the study of cTnI values in both the serum and pericardial fluid, in order to obtain the cTnI ratio. We demonstrated, for the first time, that cTnI ratio values were an independent predictor of AMI death after adjusting by confounding factors. Furthermore, we proposed a cTnI ratio valid cut-off point of 4.09 for the diagnosis of AMI. Our biomarker was found to be a valuable and highly effective tool for use in the forensic field as a complementary method to facilitate diagnosis in nonconclusive autopsies.

## Figures and Tables

**Table 1:** Baseline characteristics of included cases.

Variable	Experimental Groups			Age (mean, SD)
Cause of death (*N*, %)	**AMI group**	AMI deaths	48 (27.4)	65.5 ± 14.4
	**Control group**	Traumatic deaths	39 (22.3)	38.2 ± 20.0
		Asphyxiations	55 (31.4)	59.2 ± 17.1
		Other natural deaths	33 (18.9)	48.7 ± 17.0
Gender (*N*, %)		Male	144 (82.3)	
		Female	31 (17.7)	
Age (mean ± SD)			51.3 ± 18.9	
Cardiopulmonary resuscitation		Yes	47 (26.9)	
		No	128 (73.1)	
Postmortem interval (mean ± SD)			8.3 ± 4.9	

**Table 2:** (a) Kruskal–Wallis test comparison of cardiac troponin I (cTnI) concentrations in different causes of death and (b) association analyses of cTnI variables with the cause of death (linear regression).

**(a)**	**AMI**	**Asphyxiation**	**Trauma**	**Natural**	***p*-Value**
cTnI serum (ng/L)	0.46 (0.05–3.05)	0.11 (0.01–0.37)	0.49 (0.07–1.92)	0.21 (0.03–0.66)	0.186
cTnI pericardial fluid (ng/L)	4.73 (1.97–159.0)	1.73 (0.41–2.95)	1.93 (0.76–8.76)	0.90 (0.1–2.49)	0.012
cTnI ratio	26.4 (1.85–517.30)	18.14 (1.19–355.99)	3.66 (1.00–39.1)	2.71 (1.45–9.02)	0.005
**(b)**	**B-coefficient**	**Standard Error**	**t**	**95% CI for B**	***p*-Value**
cTnI serum	−6.63	4.16	−1.59	−14.85–1.58	0.113
cTnI pericardial fluid	−55.25	20.62	−2.68	−95.94–(−14.55)	0.008
cTnI ratio	−436.59	136.42	−3.20	−705.83–(−167.29)	0.002

95% CI for B = 95% confidence interval for B-coefficient; significant *p*-values are shown in bold.

**Table 3:** (a) Bivariate correlations of cTnI variables and acute myocardial infarction (AMI) death (Spearman correlations) and (b) Mann–Whitney test for cTnI concentrations in AMI diagnostic groups.

**(a)**	***r*-Value**	***p*-Value**	
cTnI serum	0.134	0.077	
cTnI pericardial fluid	0.395	**<0.001**	
cTnI ratio	0.188	**0.013**	
**(b)**	**AMI Diagnostic Group**	**Non-AMI Diagnostic Group**	***p*-Value**
cTnI serum (ng/L)	0.46 (0.05–3.06)	0.20 (0.04–0.86)	0.184
cTnI pericardial fluid (ng/L)	4.73 (1.79–159.00)	1.63 (0.34–17.50)	**0.041**
cTnI ratio	26.42 (1.84–517.31)	4.50 (1.15–47.12)	**0.030**

Significant *p*-values are shown in bold.

**Table 4:** Logistic regression for the prediction of AMI death.

Variable	Univariate		Multivariate	
	OR (95% CI)	*p*-Value	OR (95% CI)	*p*-Value
Age	1.03 (1.01–1.05)	0.001	1.04 (1.01–1.06)	0.001
Gender	0.45 (0.16–1.25)	0.127	0.46 (0.16–1.32)	0.149
cTnI ratio	1.01 (1.01–1.02)	0.020	1.01 (1.00–1.02)	0.022

Significant *p*-values are shown in bold.

## References

[1] Campuzano O., Allegue C., Partemi S., Iglesias A., Oliva A., Brugada R. (2014). Negative autopsy and sudden cardiac death. Int. J. Leg. Med..

[2] Michaud K., Basso C., D’Amati G., Giordano C., Kholová I., Preston S.D., Rizzo S., Sabatasso S., Sheppard M.N., on behalf of the Association for European Cardiovascular Pathology (AECVP) (2020). Diagnosis of myocardial infarction at autopsy: AECVP reappraisal in the light of the current clinical classification. Virchows Archiv..

[3] De Asmundis C., Brugada P. (2013). Epidemiology of Sudden Cardiac Death. Rev. Esp. Cardiol..

[4] Madea B., Musshoff F. (2007). Postmortem biochemistry. Forensic Sci. Int..

[5] Osuna E., Pérez-Cárceles M.D., Alvarez M.V., Noguera J., Luna A. (1998). Cardiac troponin I (cTnI) and the postmortem diagnosis of myocardial infarction. Int. J. Legal Med..

[6] Mann R.W., Bass W.M., Meadows L. (1990). Time since death and decomposition of the human body: variables and observations in case and experimental field studies. J. Forensic Sci..

[7] Woydt L., Bernhard M., Kirsten H., Burkhardt R., Hammer N., Gries A., Dreßler J., Ondruschka B. (2018). Intra-individual alterations of serum markers routinely used in forensic pathology depending on increasing post-mortem interval. Sci. Rep..

[8] Coe J.I. (1993). Postmortem Chemistry Update Emphasis on Forensic Application. Am. J. Forensic Med. Pathol..

[9] Belsey S.L., Flanagan R.J. (2016). Post-mortem biochemistry. Current applications. J. Forensic Leg. Med..

[10] Madea B. (2005). Is there recent progress in the estimation of the post-mortem interval by means of thanatochemistry?. Forensic Sci. Int..

[11] Palmiere C., Tettamanti C., Bonsignore A., De Stefano F., Vanhaebost J., Rousseau G., Scarpelli M.P., Bardy D. (2018). Cardiac troponins and NT-proBNP in the forensic setting: Overview of sampling site, postmortem interval, cardiopulmonary resuscitation, and review of the literature. Forensic Sci. Int..

[12] Batalis N.I., Marcus B.J., Papadea C.N., Collins K.A. (2010). The Role of Postmortem Cardiac Markers in the Diagnosis of Acute Myocardial Infarction. J. Forensic Sci..

[13] González-Herrera L., Valenzuela A., Ramos V., Blázquez A., Villanueva E. (2016). Cardiac troponin T determination by a highly sensitive assay in postmortem serum and pericardial fluid. Forensic Sci. Med. Pathol..

[14] Osuna E., Pérez-Cárceles M.D., Vieira D.N., Luna A. (1998). Distribution of biochemical markers in biologic fluids: application to the postmortem diagnosis of myocardial infarction. Am. J. Forensic Med. Pathol..

[15] Bañón R., Hernández-Romero D., Navarro E., Pérez-Cárceles M.D., Noguera-Velasco J.A., Osuna E. (2019). Combined determination of B-type natriuretic peptide and high-sensitivity troponin I in the postmortem diagnosis of cardiac disease. Forensic Sci. Med. Pathol..

[16] Turer A.T., Addo T.A., Martin J.L., Sabatine M.S., Lewis G.D., Gerszten R.E., Keeley E.C., Cigarroa J.E., Lange R.A., Hillis L.D. (2011). Myocardial Ischemia Induced by Rapid Atrial Pacing Causes Troponin T Release Detectable by a Highly Sensitive Assay: Insights From a Coronary Sinus Sampling Study. J. Am. Coll. Cardiol..

[17] Aissaoui A., Salem N.H., Zaqout A., Boughattas M., Belhaj M., Mosrati M.A., Chadly A. (2013). Cardiac troponin I and the postmortem diagnosis of myocardial damage. Ann Cardiol. Angeiol..

[18] Zhu B.-L., Ishikawa T., Michiue T., Li D.-R., Zhao D., Oritani S., Kamikodai Y., Tsuda K., Okazaki S., Maeda H. (2006). Postmortem cardiac troponin T levels in the blood and pericardial fluid. Part 1. Analysis with special regard to traumatic causes of death. Leg. Med..

[19] Sidlo J., Parrák V., Kvasnicka P., Majdan M., Sidlová H. (2008). On the use of biochemical markers in diagnostics of sudden cardiac death. Soud Lek..

[20] Thygesen K., Alpert J.S., Jaffe A.S., Chaitman B.R., Bax J.J., Marrow D.A., White H.D. (2018). Fourth Universal Definition of Myocardial Infarction (2018) [published correction appears in *Circulation*
**2018**, *138*, e652]. Circulation.

[21] Cao Z., Zhao M., Xu C., Zhang T., Jia Y., Wang T., Zhu B. (2019). Diagnostic Roles of Postmortem cTn I and cTn T in Cardiac Death with Special Regard to Myocardial Infarction: A Systematic Literature Review and Meta-Analysis. Int. J. Mol. Sci..

[22] Barberi C., van der Hondel K.E. (2018). The use of cardiac troponi T (cTnT) in the postmortem diagnosis of acute myocardial infarction and sudden cardiac death: A systematic review. For. Sci. Int..

[23] Pérez-Cárceles M.D., Osuna E., Vieira D.N., Martinez A., Luna A. (1995). Biochemical assessment of acute myocardial ischaemia. J. Clin. Pathol..

[24] Cina J., Brown D.K., Smialek J.E., Collins K.A. (2001). A rapid postmortem cardiac troponin T assay: laboratory evidence of sudden cardiac death. Am. J. Forensic Med. Pathol..

[25] Ooi D.S., Isotalo P.A., Veinot J.P. (2000). Correlation of antemortem serum creatine kinase, creatine kinase-MB, troponin I and tro-ponin T with cardiac pathology. Clin. Chem..

